# The Influence of Bt Maize Cultivation on Communities of Arbuscular Mycorrhizal Fungi Revealed by MiSeq Sequencing

**DOI:** 10.3389/fmicb.2018.03275

**Published:** 2019-01-09

**Authors:** Huilan Zeng, Wang Zhong, Fengxiao Tan, Yinghua Shu, Yuanjiao Feng, Jianwu Wang

**Affiliations:** ^1^Department of Horticulture, College of Life Science and Environmental Resources, Yichun University, Yichun, China; ^2^Department of Ecology, College of Natural Resources and Environment, South China Agricultural University, Guangzhou, China

**Keywords:** Illumina MiSeq sequencing, AMF community composition, Bt maize, canonical correspondence analysis (CCA), consecutive season cultivation, soil properties

## Abstract

The cultivation of transgenic *Bacillus thuringiensis* (*Bt*) has received worldwide attention since Bt crops were first released. Its ecological risks on arbuscular mycorrhizal fungi (AMF) have been widely studied. In this study, after cultivation for five seasons, the AMF diversity and community composition of two Bt maize varieties, 5422Bt1 (event Bt11) and 5422CBCL (event MO10), which both express Cry1Ab protein, and their isoline non-Bt maize 5422, as well as Bt straw after cultivation had been returned to subsequent conventional maize variety, were analyzed using Illumina MiSeq sequencing. A total of 263 OTUs (operational taxonomic units) from 511,847 sequenced affiliated with the AMF which belonged to Mucoromycota phylum Glomeromycotina subphylum were obtained. No significant difference was detected in the AMF diversity and richness (Shannon, Simpson, ACE, and Chao 1 indices) and community composition in rhizosphere soils and roots between Bt and non-Bt treatment revealed by NMDS (non-metric multidimensional scaling) and NPMANOVA (non-parametric multivariate analysis). Moreover, *Glomus* was the most dominant genus in all samples. Although there was no significant difference in the AMF community in roots and rhizosphere soils between the Bt and non-Bt maize treatments, total phosphorus (TP), total nitrogen (TN), organic carbon (OC), and pH were driving factors affecting the AMF community, and their composition varied between rhizosphere soils and roots during the maturity period of the fifth season. Compared to our previous study, the results were identical. In conclusion, no significant difference was observed between the Bt and non-Bt treatments, and the Illumina MiSeq method had higher throughput and higher quality read cover, which gave us comprehensive insight into AMF communities in agro-ecosystems.

## Introduction

The development and commercialization of transgenic modified plants (GMP) has revolutionized agriculture in the years since 1996. There were 26 countries that planted 185.1 million hectares in 2016. There are four types of GMPs that are classified in terms of their traits: herbicide-tolerant (HT), insect-resistant (IR), combined HT/IR, and resistant to viral diseases (James, 2016). Most transgenic IR crops are modified with gene sequences from *Bacillus thuringiensis (Bt)*, which allows them to express the crystal protein; most express the Cry1Ab protein that was initially developed to control the European corn borer (ECB), *Ostrinia nubilalis* Hubner, without using any external *Bt* or synthetic pesticide sprays (Castagnola and Jurat-Fuentes, 2012). The specificity and high relative toxicity of Bt proteins contributes to their high efficacy and environmental safety compared to synthetic pesticides. At present, Bt maize is the most widely grown Bt crop in the world and is planted in an area equivalent to 6.1 million ha (James, 2016). However, the rapid commercialization of Bt crops has resulted in ecological risks to non-target organisms. One of the organisms that has been a focus of research is arbuscular mycorrhizal fungus (AMF), which establishes symbiosis with the roots of plants.

Arbuscular mycorrhizal fungus forms mutually beneficial associations between species in the fungal subphylum Glomeromycotina (Spatafora et al., 2016; Davison et al., 2018) and the roots of 80% of vascular plants (Smith and Read, 2008). The fungi acquire carbohydrates from plants and provide phosphorus, nitrogen and other mineral nutrients to plants via an extraradical mycelium network (Powell and Rillig, 2018), especially when the presence of phosphorus is limited in the soil (Cheeke et al., 2012a). In addition, AMF have other effects on host plants, including a reduction in the harm that the invasion of soil-borne plant pathogens poses to roots (Liang et al., 2015), a reduction in the uptake of toxic heavy metals (Singh et al., 2017), the improvement of host plant water balance during heavy rain or drought conditions (Boyer et al., 2015; Zuccarini and Savé, 2016; Zhang et al., 2018), and the promotion of soil particle aggregation via the cohesive action of water-stable glycoproteins (Yang et al., 2017). Therefore, AMF have been identified as important soil organisms that can be used to assess the risks associated with GM crops.

Many excellent papers have been published that address the effects of Bt crops on soil microbial communities (Ferreira et al., 2003; Stotzky, 2004; Wu et al., 2004; Höss et al., 2008; Li et al., 2018), including the negative, neutral and positive effects. Some researchers have observed the significant negative effects of Bt crops on root colonization and the development of AMF. Turrini et al. (2004) showed that Bt 176 maize (which expresses the Cry1Ab protein) has significant negative effects on pre-symbiotic hyphae growth and the development of aspersoria. Castaldini et al. (2005) demonstrated that Bt maize straw that was plowed under soil for 4 months was still able to affect mycorrhizal establishment by indigenous AMF. The above mentioned studies were conducted in solo experimental conditions; Cheeke et al. (2012b) performed a field experiment that compared nine types of transgenic Bt maize with their corresponding near-isogenic parental lines in greenhouse microcosms, which demonstrated that the Bt maize plants had lower levels of AMF colonization in their roots than did the non-Bt parental lines. Similar phenomena were observed by Nagar et al. (2014), who found that DAS-59122-7 Bt maize had a negative effect on the initial development of AMF under field conditions. Other studies (Chen et al., 2016, 2017) hypothesized that the appressorium density, colonization intensity and arbuscule abundance were lower in Bt cotton roots (Jin26, GK12, and Jin 44, all of which expressedCry1Ac protein) than in roots in non-transgenic isolines (Jin7, Si3, and Ji492).

Some researchers observed a neutral effect of Bt crops on AMF colonization and communities. De Vaufleury et al. (2007) found that there was a similar degree of mycorrhizal colonization in Bt maize and non-Bt maize. Tan et al. (2011) observed identical levels of colonization in Bt maize and non-Bt maize. Cheeke et al. (2014) showed that there were no differences in spore diversity, AMF colonization or root and shoot biomass in plots that were cultivated with both Bt and parental maize. The large scale characteristics of AMF communities were determined using sequencing and DNA fingerprints; Verbruggen et al. (2012b) demonstrated that no consistent differences were detected between AM fungal communities associated with GM plants and non-GM plants via 454 pyro-sequencing of terminal restriction length polymorphism (T-RFLP). Cheeke et al. (2015) observed that the percentage of AMF colonization and the diversity of AMF communities in roots did not differ between Bt and non-Bt maize using 454 pyro-sequencing of 28S rRNA genes. Other reports have shown that there is little positive effect of Bt maize on AMF colonization in Bt maize. Zeng et al. (2015) demonstrated that colonization in Bt maize was significantly greater than in non-Bt maize after five consecutive seasons of cultivation. In terms of the straw return, although no significant differences were found in Bt and non-Bt maize, little AMF colonization in Bt maize was observed in terms of the Bt maize straw return unit than in non-Bt maize (Zeng et al., 2014).

Most studies of the effects of GMPs on AMF are focused on how Bt plants affect the colonization and symbiotic development of AMF and use microscopic and DNA fingerprinting methods; little research has been performed to investigate the effect of GMPs on symbiosis and community structure in AMFs using next-generation sequencing (NGS) analysis. Advancements in NGS technologies have had a major impact in the field by enabling the examination of large numbers of samples at a greater depth. The Illumina MiSeq platform provides researchers with scalable, high-throughput, high-quality read coverage and a lower rate of erroneous sequences compared to the DNA fingerprinting method. MiSeq sequencing technology has been used to characterize AMF communities in roots and rhizosphere soils associated with conventional plants, including apple trees, maize, seepweed and couch grass, rather than genetically modified plants (Van et al., 2015; Cui et al., 2016; Zhu et al., 2016), which demonstrates that the use of MiSeq sequencing technology was effective in investigating differences in AMF communities in soil and root samples.

In this study, we focus on AMF communities associated with the roots and rhizospheric soils of Bt and non-Bt maize after five seasons of cultivation, as well as conventional maize. Although microscopic studies and fingerprinting results have previously shown that there were minor or no significant differences in AMF composition and structure in Bt and non-Bt maize (Zeng et al., 2014, 2015), whether significant differences were observed in AMF community structure and composition using high-throughput sequencing methods remains unknown. Thus, this study had three objectives: (1) the determination of whether Bt protein in maize has an effect on AMF diversity and community composition using Illumina MiSeq sequencing; (2) the determination of which soil factors (soil chemical variables) drive AMF diversity and community composition due to changes in soil characteristics after five consecutive seasons; and (3) the comparison of results that were published previously with results obtained using Illumina MiSeq sequencing. To accomplish these objectives, we utilized Illumina MiSeq sequencing of a fragment of the small subunit (SSU) 18S rRNA gene in AMF-associated roots and rhizosphere soils from Bt and non-Bt maize after five seasons of cultivation, as well as in conventional maize.

## Materials and Methods

### Bt Maize Varieties, Bt Protein Content, AMF Colonization, and Soil Characteristics

Two Bt maize varieties, 5422Bt1 and 5422CBCL, both of which expressed Cry1Ab, and the conventional (non-Bt) isoline 5422 were planted for five consecutive seasons (from September 2009 to December 2011) according to a randomized, complete block design in a greenhouse at a site located in the Agricultural Experiment Station (23°08′ N, 113°15′ E) of South China Agricultural University in Guangzhou, China that has been previously described by Zeng et al. (2015). After five continuous seasons of cultivation, conventional non-Bt maize 5422 was cultivated on soils with added straw from Bt and non-Bt maize according to a method described by Zeng et al. (2014). Based on the colonization status of roots sampled from the conventional maize, which was determined as previously described (Zeng et al., 2014), the rate was no less than that in roots sampled during the large bell and maturity periods; thus, seedling roots in conventional maize that were sampled during the seedling period were included in this study (Zeng et al., 2014). Rhizosphere soils and roots were considered to constitute a zone that was rich in AMF. Thus, the rhizosphere soil and root samples from the maturity period during the fifth season of cultivation and the seedling period of the conventional maize were analyzed to accurately compare the results and methods used during this study. The roots were rinsed with running water and stored at -80°C prior to extraction of the nucleic acids. The rhizosphere soils were collected as follows: first, the small clumps of soil that adhered to the roots were removed. Second, the roots were oscillated in 0.85% sodium chloride at 200 rpm for 20 min. Third, the soils were collected by centrifugation at 4000 rpm for 5 min.

The soil characteristics varied during the cultivation of Bt maize. In September 2009, the soil that was used for planting of the first seasonal maize contained 34.85 g kg^-1^ organic carbon, 1.16 g kg^-1^ total N, 1.28 g kg^-1^ total P, and 19.42 g kg^-1^ total K and had a pH of 6.0. The soil changed after five consecutive seasons of cultivation; in December 2011, it contained 22.69 g kg^-1^ organic carbon, 1.13 g kg^-1^ total N, 1.82 g kg^-1^ total P, and 3.94 g kg^-1^ total K and had a pH of 5.28.

### DNA Extraction and PCR Amplification

Genomic DNA was extracted from the root samples using the cetyltrimethyl ammonium bromide (CTAB) method, and total genomic DNA was extracted from soil samples using the FastDNA SPIN Kit for Soil (Menlo Park, CA, United States) according to the manufacturer’s protocol. The final DNA concentration and purity were determined at 260/280 nm using a NanoDrop 2000 UV-vis spectrophotometer (Thermo Scientific, Wilmington, DE, United States), and the quality of the genomic DNA was verified using gel electrophoresis with 1% agarose gels.

Nested PCR was conducted to improve the specificity of the fragments. The first PCR amplification was performed using a combination of the AM fungal-specific primers AML1 (5′-ATCAACTTTCGATGGTAGGATAGA-3′) and AML2 (5′-GAACCCAAACACTTTGGTTTCC-3′) (Xu et al., 2017) with a thermal cycling program consisting of a 3 min initial denaturation at 95°C, 32 cycles of amplification (95°C for 30 s, 55°C for 30 s, and 72°C for 45 s), followed by a 10 min extension at 72°C. Using the PCR products as the template, the AM fungal-specific primers AMV4.5NF (5′-AAGCTCGTAGTTGAATTTCG-3′) and AMDGR (5′-CCCAACTATCCCTATTAATCAT-3′) (Xu et al., 2017) were added and nested PCR was performed using the following program: 94°C for 3 min, followed by 30 cycles of amplification (95°C for 30 s, 55 C for 30 s, 72°C for 45 s) and a final extension step (72°C for 10 min). Both PCR reactions were performed in triplicate in a total volume of 20 μL that contained 4 μL of 5x FastPfu Buffer, 2 μL of 2.5 mM dNTPs, 0.4 μL of each primer (10 μM), 0.4 μL of FastPfu Polymerase, 1 μL template DNA (approximately 10 ng), 0.2 μL of BSA, and 10.8 μL of ddH_2_O. An 8-bp sequence barcode was added as a tag to distinguish the PCR products from one another.

### Illumina MiSeq Sequencing and Bioinformatics Analysis

Amplicons were extracted from 2% agarose gels and purified using the AxyPrep DNA Gel Extraction Kit (Axygen, United States) according to the manufacturer’ s instructions and quantified using QuantiFluor^TM^-ST (Promega, United States). Purified amplicons were pooled at equimolar concentrations and paired-end sequenced (2 × 250) on an Illumina MiSeq platform (Illumina, San Diego, CA, United States) according to the standard protocols used at Majorbio Bio-Pharm Technology, Co., Ltd., Shanghai, China.

Based on the overlapping relationship between paired-end reads, the paired reads were merged using FLASH (Mago and Salzberg, 2011) into a sequence with a minimum overlapping length of 10 bp. The maximum error ratio of the overlap was 0.2, which filtered out reads of low quality. The obtained sequence data was first subjected to strict quality control using Trimmomatic Software (Bolger et al., 2014), including (1) the filtering of sequences with a tail quality score below Q20 using a 50 bp sliding window; (2) the filtering of sequences that were shorter than 50 bp and had a quality value larger than Q20; and (3) the filtering of sequences with a mismatch greater than zero in the barcode region (two mismatches were allowed in the primer sequences). The remaining sequences were aligned firstly against the NCBI database to remove sequences not belonging to the AMF.

The OTUs (operational taxonomic units) were assigned, which was followed by the extraction of non-repeat sequences, the removal of unrepeatable singletons, and the identification and removal of potential chimeric sequences using USEARCH 7.0 and Uclust as classification methods (Edgar, 2013). OTU picking was performed using the algorithm *cdhit* with 97% similarity as the threshold to cluster the sequences into OTUs using QIIME software (Caporaso et al., 2010). Any sample containing less than 200 reads after OTU picking was removed. The most abundant sequence in each OTU was selected as the representative sequence for that OTU, which was then aligned against the MaarjAM database (Maarjam 081, Öpik et al., 2010) to assign the taxonomic data. Taxonomy was assigned using the RDP classifier at 97% of similarity with the Bayesian algorithm, and any remaining sequences with less than 0.7 confidence in their assignment were removed (Xu et al., 2017). The database was further examined to remove sequences similar to those from chloroplasts; a total of 514,876 clean reads ranging from 221 to 240 bp were obtained from 36 samples. The reads were deposited and are available in the NCBI Sequence Read Archive (SRA) database (Accession No. SRP148734).

### Statistical Analysis

Species rarefaction curves were produced to determine if the number of samples was adequate. Based on the abundance of OTUs per sample and the Bray–Curtis distances, a series of AM community-related analyses was then performed. The diversity (Shannon and Simpson indices) and richness (Chao1 and ACE indices) were determined for the samples. Statistical calculations and data analysis were conducted using the SPSS 20 statistical software package (IBM, United States). The values are presented as the mean ± standard error (SE). Data were analyzed using one-way ANOVA, and Duncan’s multiple range test (*P* < 0.05) was used to compare the means obtained for each variable.

A Venn diagram was produced to show the unique and shared OTUs present in the AMF communities in roots and rhizosphere soils. Both constrained and unconstrained analyses were used to analyze the differences and similarities among the samples. Co-occurrence analyses was conducted to show the genera, species and taxon which co-existed in different conditions and samples, which further explained the formation mechanism of community differences between samples. Canonical correspondence analysis (CCA), which is also known as constrained analysis, was performed using R (R Core Team, 2018) to detect the major gradients in the explanatory variables, including the Bt protein and environmental variables (soil pH, soil organic carbon, total phosphorus, total nitrogen, and total potassium). Non-metric multidimensional scaling (NMDS) was also conducted to determine the differences and similarities among the AMF communities in roots and rhizosphere soils associated with sample types or maize species. Furthermore, NMPANOVA (non-parametric multivariate analysis, also known as PERMANOVA or Adonis) was conducted to further confirm significant associations between two or more groups, including the maize species (5422Bt1, 5422CBCL and 5422) and sample types (roots and rhizosphere soils). Non-metric multidimensional scaling and NMPANOVA analysis were performed using PAST (Hammer et al., 2001).

## Results

### Sequencing Information and Sampling Intensity

**FIGURE 1:**
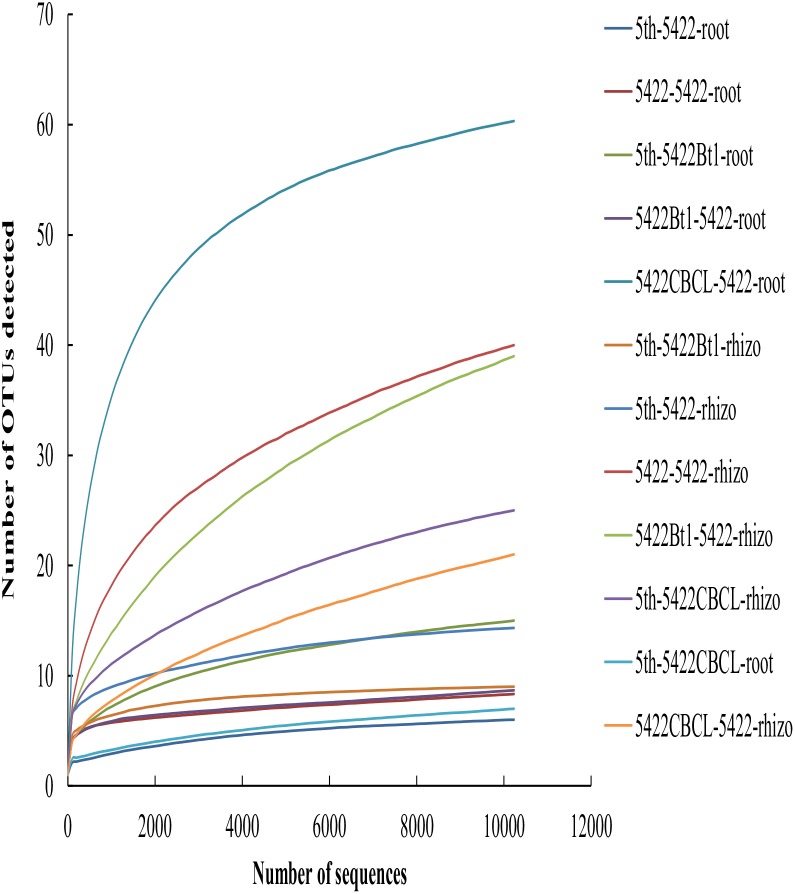
Taxa accumulation curves of AMF colonizing in roots and rhizosphere soils in all samples. The legend was labeled as follows: the fifth season or straw returning of maize variety -maize variety-sample type. The fifth season or straw returning of maize varieties including 5422Bt1, 5422CBCL and 5422; sample types including roots and rhizosphere soils. Rhizo, rhizosphere soils.

A total of 514,876 sequences ranging from 221 to 240 bp (the length of most sequences was 235 bp) were obtained from all 36 samples from Illumina MiSeq sequencing after quality control. Based on 97% similarity, a total of 263 OTUs were detected. 99.41% of all sequences (in total 511,847 sequences) belonged to Glomeromycotina subphylum, including *Glomus*, *Paraglomus, Acaulospora* and the unclassified *Glomeromycetes*. The species accumulation curves (Figure 1) tended to reach saturation plateau with increasing sample number, which indicated that the sampling intensity was sufficient.

### AMF Community Diversity and Composition

Both the diversity (Simpson and Shannon) and richness (ACE and Chao 1) indices for the OTUs from AMF communities were determined for all samples (Table 1). All of the rarefaction curves tended to reach saturation (Supplementary Figure 1), revealing that the data volume of the sequenced reads was sufficient. No significant differences in the diversity and richness of the AMF communities in the roots or rhizosphere soils of Bt maize varieties and non-Bt maize variety were found. The results indicated that the presence of the cry1Ab protein in maize did not affect the diversity and richness of the AMF community in roots and rhizosphere soils.

**Table 1:** Richness estimators and diversity indices of AMF communities sampled at the maturity period of the fifth season Bt and non-Bt maize varieties and seedling period of the subsequently conventional maize variety.

Sampling time	Sample type	Varieties	Ace	Chao	Shannon	Simpson
5^th^ season	Roots	5422	5.56 ± 1.37a	5.33 ± 1.20a	0.41 ± 0.40a	0.77 ± 0.23a
		5422Bt1	17.59 ± 9.39a	20.17 ± 8.49a	0.83 ± 0.50a	0.58 ± 0.22a
		5422CBCL	16.81 ± 9.63a	8.67 ± 2.40a	0.51 ± 0.51a	0.75 ± 0.25a
	Rhizo	5422	11.91 ± 5.96a	14.56 ± 2.30a	1.48 ± 0.21a	0.28 ± 0.05a
		5422Bt1	11.08 ± 1.08a	9.83 ± 0.44a	0.97 ± 0.29a	0.50 ± 0.12a
		5422CBCL	29.73 ± 15.39a	27.24 ± 14.01a	1.25 ± 0.37a	0.41 ± 0.14a
Subsequent conventional season	Roots	5422-5422	8.56 ± 5.11a	8.50 ± 1.04a	0.86 ± 0.18a	0.52 ± 0.09a
		5422Bt1-5422	12.88 ± 10.94a	9.00 ± 2.52a	0.90 ± 0.19a	0.50 ± 0.07a
		5422CBCL-5422	74.61 ± 52.07a	66.83 ± 57.35a	1.38 ± 0.65 a	0.46 ± 0.11 a
	Rhizo	5422-5422	70.97 ± 24.51a	52.83 ± 29.64a	1.13 ± 0.24a	0.47 ± 0.06a
		5422Bt1-5422	85.98 ± 65.57a	60.96 ± 49.48a	1.30 ± 0.17a	0.36 ± 0.07a
		5422CBCL-5422	54.08 ± 47.23 a	45.56 ± 35.63 a	0.78 ± 0.28 a	0.58 ± 0.16a


*Values are means followed by standard error. Different letters indicated statistically significant differences (*P* < 0.05) according to the Duncan’s Multiple Range Test. Rhizo, rhizosphere soils. The name of maize variety of subsequent conventional maize followed the formula: varieties of straw of maize-subsequent maize variety.*

**FIGURE 2:**
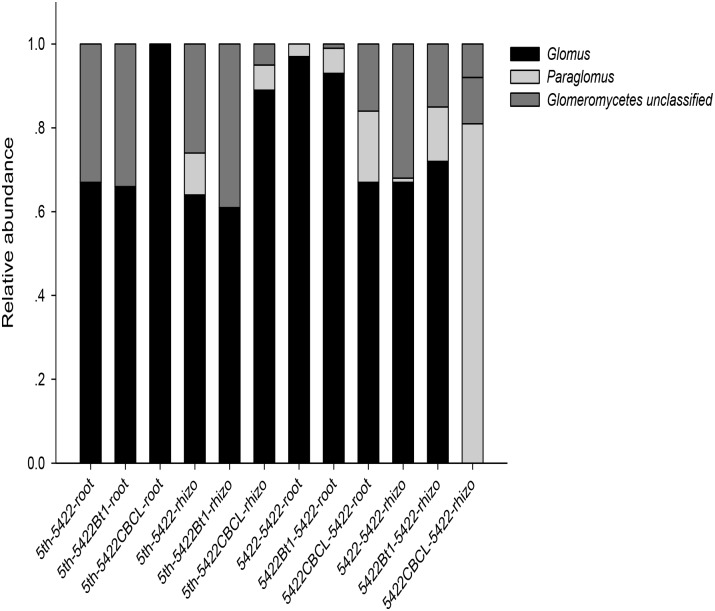
Proportional distributions of genera in the AMF community among the samples. The horizontal axes of the first six samples are labeled according to the following formula: season of cultivation-maize variety -sample type. The horizontal axes of the last six samples are labeled according to the following formula: the straw added to the subsequent maize variety–subsequent season maize variety-sample type. the fifth season or subsequent straw of maize varieties including 5422Bt1, 5422CBCL and 5422; maize types including 5422Bt1, 5422CBCL and 5422; sample types including roots and rhizosphere soils. Rhizo, rhizosphere soils.

Co-occurrence analyses suggested wide contribution of AMF in samples in virtual taxon, which showed no obvious rules between Bt and non-Bt maize varieties treatments. The results showed three genera (*Glomus, Paraglomus*, unclassified *Glomeromycetes*) including 20 virtual taxa were detected in samples harvested at the fifth season. The same three genera (*Glomus, Paraglomus*, unclassified *Glomeromycetes*) including 16 virtual taxa and *Acaulospora* genus including two virtual taxa were detected in samples harvested at the subsequent conventional season. And there were 17 and 10 virtual taxa occupied over 0.01%, respectively, which showed in Supplementary Figure 2 and the exact virtual taxa were listed in Supplementary Table 1. At the fifth season, 7, 4, and 3 AMF virtual taxa were detected in roots of 5422Bt1, 5422CBCL and 5422. And 4, 3, and 9 AMF virtual taxa were detected in soils of 5422Bt1, 5422CBCL and 5422. At the subsequent conventional season, there were 7, 6, and 7 AMF virtual taxa inhabited in roots of the subsequent conventional maize which treated with straw of 5422Bt1, 5422CBCL and 5422. And there were 5, 7, and 4 AMF virtual taxa inhabited in soils which treated with straw of 5422Bt1, 5422CBCL and 5422. In addition, in order to compare the results among our previous study, the co-occurrence results were also analyzed to the compositions and proportions on genus level in each sample (Figure 2), in which the *Acaulospora* was ignored because of its proportion was not larger than 0.01%.

*Glomus* was the dominant genus in the whole experiment, but the compositions and proportions changed (Figure 2). The number of genera was larger in soil samples than in root samples, indicating that the host has a preference for which genus colonizes it. Furthermore, although there was no significant difference, the number of genera was slightly larger (four versus three) in samples of subsequently conventional maize than those in samples cultivated for five consecutive seasons.

**FIGURE 3:**
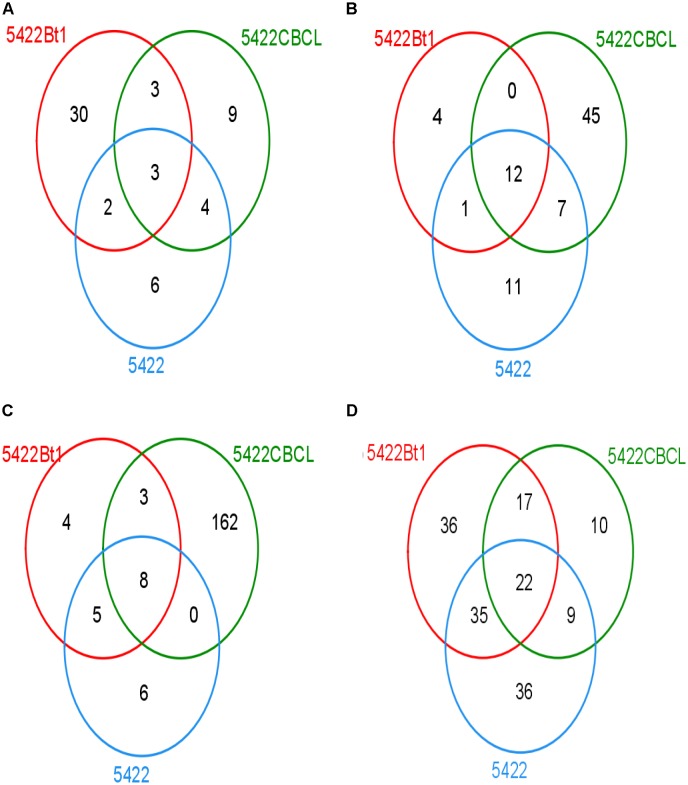
Venn diagram depicting OTUs that are shared or unique in root and soil samples. **(A)** Venn diagram of OTUs detected in roots of maize varieties cultivated for consecutive five seasons. **(B)** Venn diagram of OTUs detected in rhizosphere soils of maize varieties cultivated for consecutive five seasons. **(C)** Venn diagram of OTUs detected in roots of conventional maize variety cultivated with straw of the 5^th^ maize varieties. **(D)** Venn diagram of OTUs detected in rhizosphere soils of conventional maize variety cultivated with straw of the 5^th^ maize varieties.

The Venn diagram showed that different samples had different OTU composition (Figure 3). At the fifth season sampling, there were 57 OTUs in roots, 80 OTUs in rhizosphere soils. At the subsequent conventional maize sampling, there were 188 OTUs in roots and 155 OTUs in rhizosphere soils. There were 30, 9, and 6 unique OTUs in the roots of 5422Bt1, 5422CBCL and 5422 of maize cultivated for five consecutive seasons, respectively. Only three OTUs of AMF were shared in roots of 5422Bt1, 5422CBCL and 5422 of maize cultivated for five consecutive seasons. The numbers of unique OTUs of AMF in rhizosphere soils of 5422Bt1, 5422CBCL and 5422 maize, which cultivated for five consecutive seasons, were 4, 45, and 11, respectively. However, the OTUs of AMF were more abundant in subsequent conventional maize roots and rhizosphere soils than those in Bt and non-Bt maize cultivated for five consecutive seasons. In roots of subsequent conventional maize, the numbers of unique OTUs of AMF associated with straw return of 5422Bt1, 5422CBCL, and 5422 were 4, 162, 6, respectively. The large number of unique OTUs indicated the wide range of genetic diversity of AMF in subsequent conventional maize root associated with a straw return of 5422CBCL. There were only eight OTUs shared in roots of subsequent conventional maize associated with a straw return of 5422Bt, 5422CBCL, and 5422, respectively. In rhizosphere soils of subsequent conventional maize, the number of unique OTUs of AMF associated with straw return of 5422Bt1, 5422CBCL, and 5422 was 36, 10, 36, respectively. There were 22 OTUs shared in rhizosphere soils of subsequent conventional maize associated with a straw return of 5422Bt, 5422CBCL, and 5422.

### Relationships Among AM Fungus Parameters and Soil Factors

**FIGURE 4:**
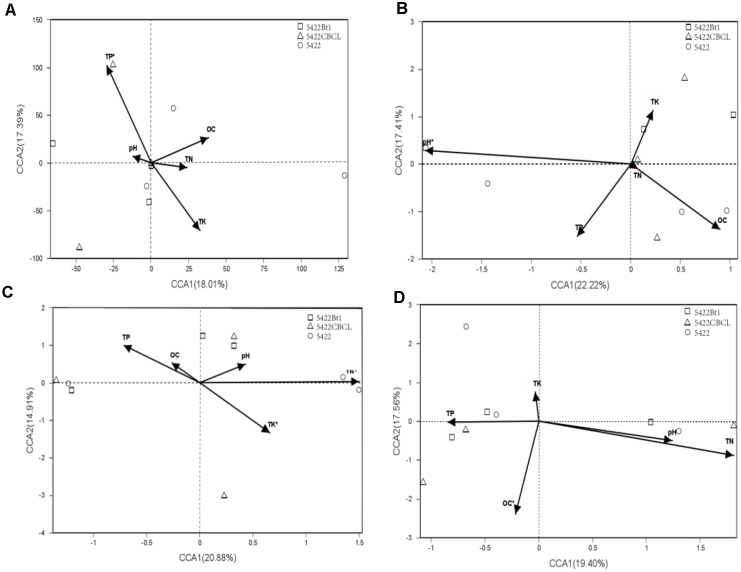
Canonical correspondence analyses (CCA) biplot showing the relationship between the detected varieties and soil characteristics in samples. **(A)** Roots of maize varieties cultivated at the 5^th^ season. The related eigenvalues were as follows: DCA1 = 1.1976, DCA2 = 15.8373, DCA3 = 2.9401, DCA4 = 7.0103. pH:P = 0.902, OC:*P = 0.506*, TN:*P = 0.768*, TP:***P = 0.001***, TK:*P = 0.096.* **(B)** Rhizosphere soils of maize varieties cultivated at the 5^th^ season. The related eigenvalues were as follows: DCA1 = 4.8569, DCA2 = 3.264, DCA3 = 1.83, DCA4 = 1.83, ***pH:P = 0.003***, OC:*P = 0.12*,TN:*P = 0.99*, TP:*P = 0.179*, TK:*P = 0.431.* **(C)** Roots of subsequent conventional maize variety. The related eigenvalues were as follows: DCA1 = 10.2588, DCA2 = 1.9029, DCA3 = 1.2665, DCA4 = 0.783.pH:*P = 0.662*, OC:*P = 0.698*, ***TN:P = 0.001***, TP*:P = 0.122*, ***TK:P = 0.03.*** **(D)** Rhizosphere soils of subsequent conventional maize variety. The related eigenvalues were as follows: DCA1 = 6.2066, DCA2 = 3.4876, DCA3 = 3.1145, DCA4 = 3.3648. pH:*P = 0.468*, *OC****:P = 0.017*,** TN:*P = 0.113*, TP:*P = 0.737*, TK:*P = 0.794*.

Based on the results of detrended correspondence analysis (DCA), one of the eigenvalue was larger than the number of four, which indicated CCA ordination analysis was more suitable for the samples. The CCA ordination analysis of the AMF community in roots and rhizosphere soils were performed using total nitrogen (TK), total phosphorus (TP), total potassium (TK), organic carbon (OC), and pH as explanatory variables. The results showed large variations in different sampling times and sample types. The biplot of CCA result with interspecies of Bt and non-Bt maize treatments distances is shown in Figure 4. In roots of Bt and non-Bt maize cultivated during the fifth season, the significant environmental correlation to the AMF community variation was retrieved by total phosphorus (*P* = 0.001). In rhizosphere soils of Bt and non-Bt maize cultivated during the fifth season, pH was the environmental factors significantly affecting the AMF community (*P* = 0.003). Total nitrogen (*P* = 0.001) and total potassium (*P* = 0.03) showed strongly significant correlation to the species of AMF community in roots of subsequent conventional maize. In rhizosphere soils of subsequent conventional maize, the significant correlation to AMF community variation was retrieved by organic carbon (*P* = 0.017).

**FIGURE 5:**
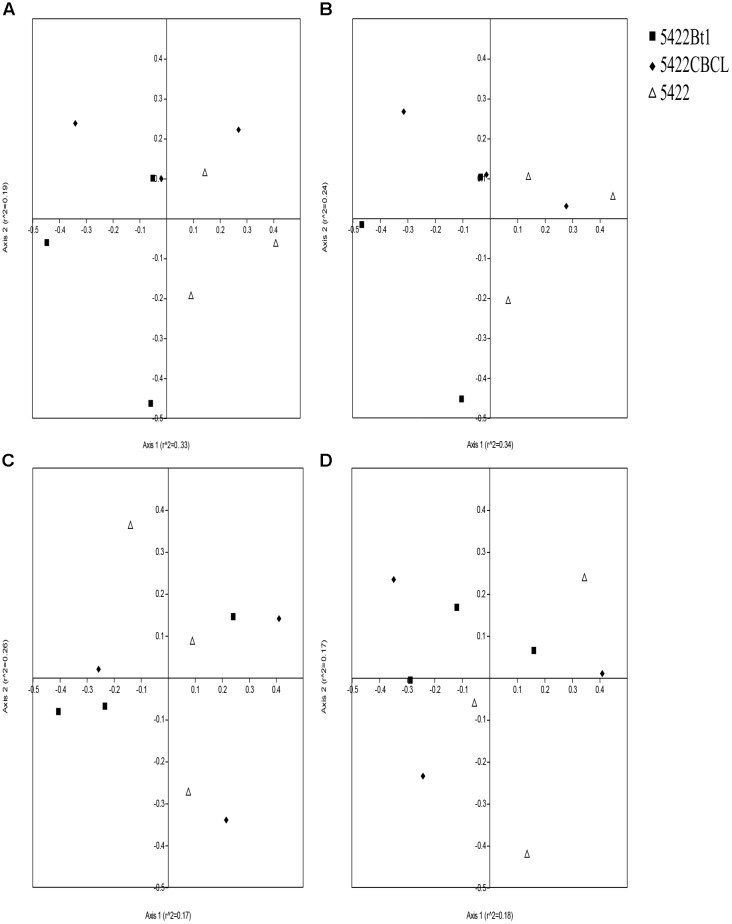
Non-metric multidimensional scaling (NMDS) ordination plots of the AM fungal communities in roots **(A,C)** and rhizosphere soils **(B,D)** associated with Bt and non-Bt maize varieties cultivated for five consecutive season **(A,B)** or straw returning to subsequent conventional maize variety **(C,D)**.

Non-metric multidimensional scaling results based on Bray–Curtis distance measure showed that the data points were overlapped in the plots, which revealed that there were no statistically significant differences in the AMF community composition of the Bt and non-Bt maize or Bt maize straw treatments (Figure 5) (according to 95% confidence ellipse interval, which is shown in Supplementary Figure 3). The NPMANOVA results with 9,999 permutations further revealed that there were no significant differences in AMF community structure among roots and rhizosphere soils of Bt and non-Bt maize, nor were there significant differences between those of subsequent conventional maize associated with straw of Bt and non-Bt maize which cultivated for five consecutive seasons (Table 2). However, significant difference was observed in AMF community structure associated with rhizosphere soils and roots of Bt and non-Bt maize cultivated for five consecutive seasons (*F* = 2.005, *P* = 0.0104).

**Table 2:** Non-parametric multivariate analyses (NPMANOVA) of AMF communities in related treatments.

Sampling time	Treatment	NPMANOVA	
		
		*F*	*P*
5^th^ season	Sample type	2.005	**0.0104^∗^**
	Varieties in rhizo	0.9044	0.5391
	Varieties in roots	0.8647	0.6924
Subsequent conventional	Sample type	0.7922	0.5921
	Varieties in rhizo	0.7795	0.787
	Varieties in roots	0.7073	0.8087


*The bold and ‘^∗^’ indicated significant differences (*P* < 0.05), varieties including 5422, 5422Bt1 and 5422CBCL, sample types including roots and rhizospheric soils. Rhizo, Rhizosphere soils.*

## Discussion

The sequences detected using the primer set AMV4.5NF/AMDGR belonged to *Glomeromycota*, which occupied 99.41%. Our PCR program enhanced the specificity of AMF compared to several other studies using the same primer set, AMV4.5NF/AMDGR (Bainard et al., 2015; Liu et al., 2017; Xu et al., 2017). The reason was that nest PCR improved the specificity of AMF in our study. We obtained nearly 514,876 AMF sequences, forming 263 OTUs in all treatments, which was higher than those previously published by our team (Zeng et al., 2015). Only 29 OTUs were detected in our previous study of rhizosphere soils of maize cultivated for five consecutive seasons, which used a cloning method, while 80 OTUs were detected in this study of rhizosphere soils of maize cultivated for five consecutive seasons. Larger significant differences were detected in roots of subsequently conventional maize. Only 47 OTUs were detected in our previous study in roots of subsequently conventional maize associated with straw of Bt maize varieties, while 188 OTUs were detected in this study in roots of subsequently conventional maize associated with straw of Bt maize varieties. Moreover, the number of OTUs obtained was higher than that found in other sequencing efforts from other ecosystem (Dumbrell et al., 2011; Zhu et al., 2016). The causes of these results were that (1) the Illumina MiSeq sequencing had higher sequencing depth and throughput, as well as higher quality read cover, which gave us a comprehensive insight into AMF community in agro-ecosystem in this study, and (2) the genetic fragments amplified by primers had differences assigned to OTUs.

Diversity of AMF indicated the adaptability and stability of community, further came a play role in nutrient uptake, resistance to adverse environment and so on (Parniske, 2008; Smith and Read, 2008). Although differences in the number of OTUs were detected between results of MiSeq sequencing and Sanger sequencing based on the same samples, the richness (ACE and Chao 1) and diversity (Shannon and Simpson indices) of AMF community were not significant different between Bt and non-Bt maize cultivation or straw returning, which was the same in statistics as results published previously in our team (Zeng et al., 2014, 2015). Combining these results with OTUs assign indicated that the method T-RFLP and cloning detected by Sanger sequencing also had high accurate to detect the difference in AMF community diversities between Bt and non-Bt maize in root and soil samples.

Although many researchers have reported that soil physical and chemical characteristics had impacts on AMF diversity, there was no consistent conclusion. Many factors were reported to drive the distribution of AMF community, including pH, Mn, Zn (Alguacil et al., 2016), available extractable Ca, K (Casazza et al., 2017), phosphorus (De Beenhouwer, 2015), nitrogen (Treseder and Allen, 2002), and soil organic carbon (Yang et al., 2011). According to our results shown by CCA biplots (Figure 4), total phosphorus (TP) was the main factor driving the AMF community in roots of maize cultivated for consecutive seasons, and pH was considered as the main factor driving the AMF community in rhizosphere soils of maize cultivated for consecutive seasons. These results were in accordance with results published by De Beenhouwer (2015), which reported that pH, phosphorus availability were significantly varied AMF community composition. Interestingly, the AMF community in rhizosphere soils and roots of subsequent conventional maize was driven by total nitrogen (TN) and organic carbon (OC) rather than total potassium (TK, which showed significant lower concentration in soils detected at the fifth season compared to that detected at the first season), which was explained by the straw decomposition (used as organic manure in traditional agriculture) of Bt and non-Bt maize as main factor increasing concentrations of total nitrogen and organic carbon (Zhu et al., 2014, 2016). However, there was no significant difference in the AMF community between Bt and non-Bt maize treatments, shown by results of NMDS and NPMANOVA (Figure 5 and Table 2), indicating Bt maize consecutive cultivation and straw returning did not affect the AMF community in soils and rhizosphere soils. The reasons of the results may be that AMF was affected by multiple factors, not only soil chemicals but also other factors such as host plant and climate changes (Oehl et al., 2003; Wu et al., 2004; Alguacil et al., 2008; Shi et al., 2015).

The number of genera was slightly larger in soil samples than those in root samples, which in accordance with results showed by Davison et al. (2011), indicated that AMF in rhizosphere soils could be considered a pool of species, a fraction of which was colonized by plants. Furthermore, although there was no significant difference, the number of genera was larger in samples of subsequently conventional maize than those in samples of cultivated for five consecutive seasons, which can be explained by the straw returning of Bt maize that may change the AMF community composition slightly.

In the present study, 20 and 18 virtual taxa were detected in the fifth season and subsequent conventional season, respectively. The existence of multiple virtual taxa suggested relative high diversity and wide distribution of AMF in each sample. Because of the species is virtual according 97% sequence similarity, the analysis in the genus level regarded as more comprehensive were also conducted. There were three main genera detected, including *Glomus*, *Paraglomus*, and unclassified *Glomeromycetes*, which were different from those genera previously detected by cloning. *Glomus* and *Paraglomus* were abundant genera in soil and root samples in natural ecosystems, which often were detected in ecosystems such as agricultural and impacted semiarid areas (Lee et al., 2006; Mergulhão et al., 2010). *Rhizophagus* was detected in roots of 5422 and rhizosphere soils of 542Bt1 cultivated for consecutive seasons in our previous study, but they were not detected in the same samples in the present study. Moreover, one more genus (*Funneliformis*) was detected in the roots and rhizosphere soil samples of subsequently conventional maize by cloning method in our previous study than composition of AMF genera detected in the present study. The larger amplified fragments aimed at SSU-ITS-LSU regions previously may be the main reason leading to the results. The *Rhizophagus* and *Funneliformis* genus was also a common genus of AMF that had been detected in diverse host species and environments with the development of molecular techniques compared to traditional spore screening technique (Oehl et al., 2009; Verbruggen et al., 2012a; Symanczik et al., 2014). However, *Glomus* was the dominant genus in roots and rhizosphere soils, in accordance with results of other articles (Oehl et al., 2009; Zarei et al., 2010; Verbruggen et al., 2012a; Liu et al., 2017). The dominance of *Glomus* has been widely found in various ecosystems, such as coastal saline-alkaline land soils (Cui et al., 2016), heavy textured agricultural soils (Mathimaran et al., 2005), degraded mined soils (Albert, 2015), and agricultural soils (Dai et al., 2014). This can be explained by the characteristics of *Glomus*, which can easily survive and reproduce via mycelium, spores or fragments of colonized plants. Further, *Glomus* could be more resistant to disturbances and stresses in the environment often enough to play an important role in performing ecological function (Daniell et al., 2001).

Although many studies were conducted to study the composition and structure of AMF communities in roots and rhizosphere soils using Illumina MiSeq (Liu et al., 2017; Xu et al., 2017; Yang et al., 2017), few studies focused on the effect of Bt maize cultivation and straw returning on the composition and structure of AMF communities. Moreover, several studies emphasized the long-term effect of GM crops on non-target microorganism (Barriuso et al., 2012; Hannula et al., 2012; Zeng et al., 2015), which did not use the MiSeq sequencing. In addition, our results were in accordance with Barriuso et al. (2012) and Hannula et al. (2012), which reported that the genetically modified trait had no or minor effects on rhizobacterial communities or AMF communities.

Assessing the effects of genetically modified plants on non-target organisms is a long-term process. It needs to be studied from multiple aspects such as nutrient cycling, glomalin concentrations and responses in roots and soils and Bt protein fingerprints in the whole cultivation of Bt maize. Our studies will pay further attention to examining the responses of nitrogen and carbon cycling mediated with AMF.

## Conclusion

The data from our study revealed that 5422Bt1 (event Bt11), and 5422CBCL (MON810) cultivation for five consecutive seasons and straw returning to soils had no significant effect on AMF community diversity and structure in rhizosphere soils and roots of subsequently conventional maize 5422. Moreover, the soil characteristics of total phosphorus (TP), total nitrogen (TN), organic carbon (OC) and pH were driving factors affecting the AMF community in different samples. Compared with our previously published data, the Illumina MiSeq method had higher throughput and higher quality read cover, which gave us a comprehensive insight into the AMF community in agro-ecosystems.

## Author Contributions

JW designed the experiments. WZ performed the majority of the experiments. YS supervised the performance of the experiments. YF contributed to gain reagents, materials, and analysis tools. WZ, HZ, and JW analyzed the data. HZ and FT wrote the main manuscript text. All authors reviewed the manuscript.

## Conflict of Interest Statement

The authors declare that the research was conducted in the absence of any commercial or financial relationships that could be construed as a potential conflict of interest.
