# Supplementary material for: The Influence of Bt Maize Cultivation on Communities of Arbuscular Mycorrhizal Fungi Revealed by MiSeq Sequencing
Source: Front Microbiol. 2019 Jan 9;9:3275. doi: 10.3389/fmicb.2018.03275 (PMC6334669; doi:10.3389/fmicb.2018.03275)
Supplement: Supplementary file 2 [file Data_Sheet_1.docx]

Supplementary Material

The influence of Bt maize cultivation on communities of arbuscular mycorrhizal fungi revealed by MiSeq sequencing

**Huilan Zeng, Wang Zhong, Fengxiao Tan, Yinghua Shu, Yuanjiao Feng, Jianwu Wang ***

**Correspondence:** Corresponding Author: wangjw@scau.edu.cn

**Supplementary Figure 1** Accumulation curves of AMF community diversity in roots and rhizosphere soils in all samples.

The legend was labeled as follows: the fifth season or straw returning of maize variety-maize variety-sample type. The fifth season or straw returning of maize varieties including 5422Bt1, 5422CBCL and 5422; sample types including roots and rhizosphere soils. Rhizo=Rhizosphere soils.

**Supplementary Figure 2** The co-occurrence network analyses in samples. (A) showed the genus level of AMF in samples harvested at the fifth season; (B) showed the genus level of AMF in samples harvested at the subsequent conventional season; (C) showed the taxon level of AMF in samples harvested at the fifth season; (D) showed the taxon level of AMF in samples harvested at the subsequent conventional season.

(A)

(B)

(C)

(D)

**Supplementary Figure 3** Nonmetric multidimensional scaling (NMDS, which shows 95% confidence ellipse) ordination plots of the AM fungal communities in roots (A, C) and rhizosphere soils(B, D) associated with Bt and non-Bt maize varieties cultivated for five consecutive season (A,B) or straw returning to subsequent conventional maize variety (C,D).

The pink filled square represents 5422Bt1, the gray rhombus represents 5422CBCL, and the light blue triangle represents 5422 treatment.
